# Dignity therapy intervention fidelity: a cross-sectional descriptive study with older adult outpatients with cancer

**DOI:** 10.1186/s12904-021-00888-y

**Published:** 2022-01-11

**Authors:** Tasha M. Schoppee, Lisa Scarton, Susan Bluck, Yingwei Yao, Gail Keenan, Virginia Samuels, George Fitchett, George Handzo, Harvey M. Chochinov, Linda L. Emanuel, Diana J. Wilkie

**Affiliations:** 1 University of Florida College of Nursing, Gainesville, Florida USA; 2 Center for Palliative Care Research & Education, Gainesville, Florida USA; 3 Community Hospice & Palliative Care, Jacksonville, Florida USA; 4 University of Florida Department of Psychology, Gainesville, Florida USA; 5 Rush University Medical Center, Chicago, IL USA; 6 HealthCare Chaplaincy Network, New York, NY USA; 7 CancerCare Manitoba, Winnipeg, Manitoba Canada; 8 Northwestern University, Evanston, IL USA

**Keywords:** Intervention Fidelity, Dignity therapy, Palliative care

## Abstract

**Objectives:**

Intervention fidelity is imperative to ensure confidence in study results and intervention replication in research and clinical settings. Like many brief protocol psychotherapies, Dignity Therapy lacks sufficient evidence of intervention fidelity. To overcome this gap, our study purpose was to examine intervention fidelity among therapists trained with a systematized training protocol.

**Methods:**

For preliminary fidelity evaluation in a large multi-site stepped wedge randomized controlled trial, we analyzed 46 early transcripts of interviews from 10 therapists (7 female; 7 White, 3 Black). Each transcript was evaluated with the Revised Dignity Therapy Adherence Checklist for consistency with the Dignity Therapy protocol in terms of its Process (15 dichotomous items) and Core Principles (6 Likert-type items). A second rater independently coded 26% of the transcripts to assess interrater reliability.

**Results:**

Each therapist conducted 2 to 10 interviews. For the 46 scored transcripts, the mean Process score was 12.4/15 (SD = 1.2), and the mean Core Principles score was 9.9/12 (SD = 1.8) with 70% of the transcripts at or above the 80% fidelity criterion. Interrater reliability (Cohen’s kappa and weighted kappa) for all Adherence Checklist items ranged between .75 and 1.0. For the Core Principles items, Cronbach’s alpha was .92.

**Conclusions:**

Preliminary findings indicate that fidelity to Dignity Therapy delivery was acceptable for most transcripts and provide insights for improving consistency of intervention delivery. The systematized training protocol and ongoing monitoring with the fidelity audit tool will facilitate consistent intervention delivery and add to the literature about fidelity monitoring for brief protocol psychotherapeutic interventions.

**Supplementary Information:**

The online version contains supplementary material available at 10.1186/s12904-021-00888-y.

## Background

Intervention fidelity refers to the true and consistent adherence to implementation of an established intervention plan or protocol. In a healthcare environment, it indicates that all providers have done what they were trained to do, intended to do, and what they promised to do; they deliver all of the intervention components with authenticity. This authentic adherence, which is imperative both in research and in clinical practice to achieve desired effects, is particularly challenging to measure for communication-based interventions such as Dignity Therapy (DT) [1–3]. DT is a psychotherapeutic intervention developed by Dr. Harvey Max Chochinov with the goal of supporting the dignity of the person through a supportive conversation with a trained therapist designed to facilitate development of a legacy document that the person may share with a significant person(s) in his/her life [2]. Patient outcomes across DT studies have been inconsistent [3, 4], but the reports lack sufficient evidence of intervention fidelity. Therefore, it cannot be determined whether variation in study results is related to inconsistent implementation or other factors [3–7]. The purpose of this study was to examine fidelity of DT delivered to patients with cancer receiving outpatient palliative care.

Deficits in knowledge and understanding of the importance of intervention fidelity have been cited as barriers to treatment adherence even among experienced researchers [5]. Verifying fidelity enables confidence that conclusions drawn are valid [6]. Failure to ensure adherence to an efficacious intervention increases the risk that impact is not realized, yet without clarity of the cause. Achieving adequate intervention fidelity is evidence of effective operationalization; if an intervention implementation can be demonstrated to have reached consistency among interventionists and to the intervention protocol, it indicates that the process is effective. It is important to understand that this fidelity is very different from routinization, in which there may be superficial adherence to an intervention, while the deeper imperatives of the intervention are missed. For DT, these deeper imperatives are the components of dignity as Chochinov identified [8]. If the participant’s dignity is not actively supported, the protocol becomes a mere list of questions (Table 1) that an individual could read and answer in isolation. Conversely, deviating too far from the content the questions are designed to elicit may make it hard to produce a legacy document from the interaction. As DT is a brief psychotherapy intended to support the dignity of the person and provide a legacy document, ensuring fidelity to the intended therapist-participant interaction facilitates optimal outcomes.

**Table 1:** Dignity Therapy Protocol Questions [9]

1. Tell me a little about your life history, particularly the parts that you either remember most or think are the most important? When did you feel most alive?	
2. Are there specific things that you would want your family to know about you, and are there particular things you would want them to remember?	
3. What are the most important roles you have had in life (e.g., family roles, vocational roles, community-service roles)? Why were they so important to you and what do you think you accomplished in those roles?	
4. What are your most important accomplishments, and what do you feel most proud of?	
5. Are there particular things that you feel still need to be said to your loved ones or things that you would want to take the time to say once again?	
6. What are your hopes and dreams for your loved ones?	
7. What have you learned about life that you would want to pass along to others? What advice or words of guidance would you wish to pass along to your son, daughter, husband, wife, parents, or other(s)?	
8. Are there words or perhaps even instructions that you would like to offer your family to help prepare them for the future?	
9. In creating this permanent record, are there other things that you would like included?	

The DT therapist must demonstrate vital components to foster the individual’s dignity [10]. These vital components, including the therapist’s Attitude, Behavior toward the participant, expressions of Compassion, and therapeutic Dialogue have been described by a construct entitled the ABCDs of Dignity, which is detailed in the Measures section [10]. The concepts are foundational to the DT intervention [10].

Several systematic reviews of DT studies in a variety of patient populations revealed considerable variability in outcomes. Results included reports of lack or limited impact on emotional symptoms [3, 4], spirituality [11], ,and physical and psychological symptoms [11], ,as well as inconsistency with regard to acceptability [12], However, there were also reports regarding increased sense of meaning and purpose [3, 11], ,acceptability and positive adaptability to various cultures [4], ,and improved psychological well-being [13]. One plausible explanation for these inconsistencies is that the fidelity of the DT was less than needed for an intervention effect.

In publications of DT intervention fidelity, approaches varied. In two articles, authors noted that recordings were reviewed: Bentley and colleagues reviewed 3 recordings (10%) [9] and Chochinov et al [14] had an unspecified number of recordings reviewed by the research coordinator. Four authors reported randomly selecting transcripts for review: three of these authors reviewed 25 to 33% of transcripts [15–17] and one did not specify the number of transcripts selected for review [18]. Another study was reported with 33% of 45 transcripts reviewed, but no details were given regarding the selection process [19]. Hall and colleagues reported in a separate study that they had established a quality assurance protocol, but no details were provided [20]. In the remaining four articles, the method for review was not stipulated. In those articles that did specify some review process, the authors did not provide details regarding any scoring process, other measure of fidelity, or interrater reliability of the scores.

Further, although reproducible training is imperative in preparing the DT therapists to facilitate delivery of DT with fidelity [21], there was a lack of detail in the literature regarding such preparation [22]. To support reproducibility of intervention delivery, we had developed and implemented a Refined DT Training Protocol for the DT therapists [22] who delivered DT to outpatients with cancer receiving palliative care. The specific aim of this study was to examine inter-rater reliability of the fidelity ratings for DT intervention delivery based on adherence to the process and core ABCD principles among therapists trained according to the refined training protocol.

## Methods

### Design

In this cross-sectional study that was part of a larger stepped-wedge randomized controlled trial [7], we initiated evaluation of DT implementation for fidelity to the intervention training protocol. The research design enabled us to evaluate a cross-section of transcripts, across sites and therapists, for evidence of intervention delivery fidelity. The study was approved by Institutional Review Boards at all sites.

### Setting and sample

Each DT interview was conducted in a private interview room in an outpatient clinic at an academic medical center with an outpatient palliative care clinic. Each interview was conducted by a DT therapist trained according to the Refined DT Training Protocol (Table 2) [22]. For this study, we included all intervention transcripts that were generated and available between April 2019 and July 2020. Excluded were transcripts that were incomplete due to recording errors. There was a total of 46 interview transcripts across four sites, comprised of one interview for each of the 46 adults with cancer who were 55 years of age or older and receiving outpatient palliative care. The ten DT therapists included 6 chaplains and 4 nurses who conducted the interviews from which a professional transcription service produced and verified accuracy of the 46 transcripts.

**Table 2:** Training Components

Initial Training Components Topic	Presentation Format	Time Allotted
Introduction & DT background	Lecture	1.5 h
Key elements & techniques	Discussion	1 h
DT demonstration	Video with Q & A	1.75 h
DT practice	Progressive role play with mock patient	1 h
DT demonstration	Live interview and debrief	1.75 h
Experiential role play	Role play in pairs and debrief	1 h
Overview of editing process	Case examples and discussion	1 h
Opportunities & challenges	Discussion	1.5 h
Interview with a DT recipient	Pre-recorded video	0.5 h
**Other Training Components Topic**	**Presentation Format**	
DT Textbook	Written text	
Practice Interviews	Virtual meetings with standardized patient	
General & Individual Feedback	Phone calls with DT experts	
Process Tracking	Documentation form	
DT Training Manual	Electronic document	
Quarterly Support Sessions	Virtual meetings with DT experts	

*DT* Dignity Therapy

### Instruments

#### Tracing form

The research team determined that a tool was needed to guide the DT therapists in the process of the DT intervention, supporting attention to all process-related aspects of the interview and legacy document creation. The tool would also be used by the auditors to track and evaluate fidelity to the DT protocol. To address this need, the team of experts in DT and clinical trial implementation developed the DT Contact & Process Tracking Form (Tracking Form) (Additional file 1). The Tracking Form was comprised of 4 sections that allowed the research coordinator and therapist to document each of the contacts with the study participant. More than 30 process steps were included in the 4 contacts. The information gathered via the Tracking Form provided source data from which some of the process items could be scored; it did not provide any information regarding the Core Principles items.

#### Adherence checklist

A 10-item adherence checklist had been used in previous DT studies (H. M. Chochinov, personal communication, July 30, 2018), but its reliability had not been established and there was no code book to guide its scoring. In collaboration with the original DT creator, we refined the form as the Revised DT Adherence Checklist (Adherence Checklist) and developed an accompanying Code Book to provide definitions for coding decisions. The Adherence Checklist and Code Book enumerated more fully the components of the original 10-item adherence checklist; definitions and examples were provided for coding each of the revised 21 items (Table 3). Our Adherence Checklist included items to evaluate the process (15 items, Table 4) and items to evaluate the core principles (6 items, Table 5) of the DT intervention as described in the following sections.

**Table 3:**
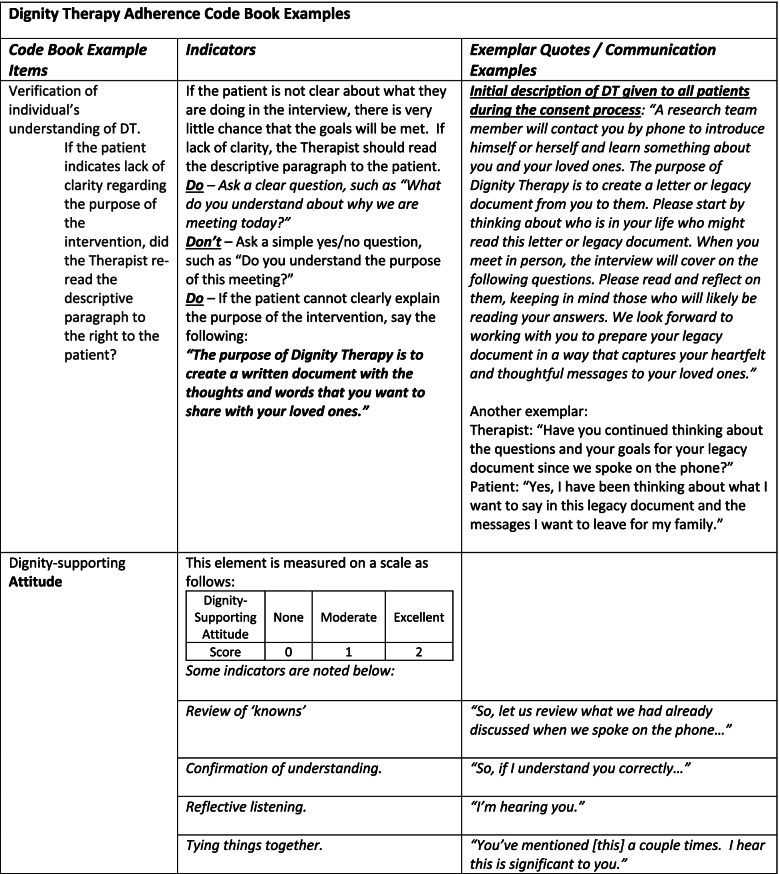
Dignity Therapy Adherence Code Book Examples

**Table 4:** Statistics Regarding DT Intervention Process (From DT Adherence Checklist) by Item

Process Item	***Frequency*** (% coded ‘Yes’^a^) (***N*** = 46)	Cohen’s Kappa (***n*** = 12)
**Verification of individual’s understanding of DT**	5 (11%)	1.0
**Questions asked per DT protocol**	42 (91%)	1.0
**Therapist’s avoidance of introducing erroneous content**	39 (85%)	1.0
**Therapist flexible to follow individual direction**	45 (98%)	1.0
**Therapist flexible to individual’s content boundaries**	45 (98%)	1.0
**Therapist consistently focused to DT goal**	25 (54%)	.75
**Therapist of neutral and supportive**	43 (93%)	1.0
**Therapist empathically attuned**	42 (91%)	1.0
**Therapist used elaborative techniques to elicit details**	39 (85%)	1.0
**Sequence of contacts as per the DT protocol**	35 (76%)	.75
**Participant prompted to designate alternate recipient**	37 (80%)	1.0
**Preferred method of document delivery verified**	42 (91%)	1.0
**Editing process in accordance with the DT protocol**	46 (100%)	1.0
**Participant given opportunity revise document**	46 (100%)	1.0
**Document read to the patient or offered to do so**	46 (100%)	1.0

*DT* Dignity Therapy

*f* frequency

*Min/Max* Minimum/Maximum

^a^ = % of time the item was met across the cases

**Table 5:** Statistics Regarding DT Core Principles (From DT Adherence Checklist) By Item

Core Principle	Mean^a^ (SD) (***N*** = 46)	Median (Min/Max) (***N*** = 46)	Weighted Kappa (***n*** = 12)
**Sense of Connectivity**	1.6 (.6)	2 (0/2)	1.0
**Overall Adherence to DT**	1.4 (.6)	1 (0/2)	.75
**Dignity-supporting Attitude**	1.6 (.6)	2 (0/2)	1.0
**Dignity-supporting Behavior**	1.9 (.4)	2 (1/2)	1.0
**Dignity-supporting Compassion**	1.8 (.4)	2 (1/2)	1.0
**Dignity-supporting Dialogue**	1.9 (.4)	2 (0/2)	1.0

*DT* Dignity Therapy

*SD* Standard Deviation

*Min/Max* Minimum/Maximum

^a^ Highest Possible Process Score per Item = 2

The 15 process items were scored dichotomously, ‘yes’ (the process element was met, assigned a score of 1) or ‘no’ (the process element was not met, assigned a score of 0). The items reflected essentials of the interview such as whether the therapist followed the framework of the protocol and whether he/she used probing questions to elicit valuable elements of the participant’s story. There are also items regarding the process, such as whether the therapist requested a designee to receive the document if the participant was not available and whether the participant was given the opportunity to make changes to the document. Appearing in Table 4 are the 15 items for the Process score, which ranges between 0 and 15. The a priori criteria of minimal fidelity was 80%, which was a score of 12.

The six items representing the therapist’s adherence to the core principles of DT, were measured on a Likert-type scale. These items focused on the overall flow and tenor of the interview as well as the therapist’s expression of the ABCDs of Dignity. The ABCDs reflect Chochinov’s model of dignity [10]: (A) is *attitude* that is subject to self-awareness and self-reflection; (B) is *behavior* that is kind, respectful, and attentive; (C) is expressed *compassion* for the suffering and experience of the individual; and (D) is *dialogue* that connects with the individual beyond the illness or frailty (Table 5) [10]. The scale for the Core Principles score was defined as: no fidelity (assigned a score of 0); moderate fidelity (assigned as 1); or excellent fidelity (assigned a score of 2). The sum of these items, the Core Principles score, could range between 0 and 12. The a priori criteria of minimal fidelity was 80%, which was a score of 10.

Content validity of the Adherence Checklist was assessed by a team of 6 experts, including the main DT creator [2]. To assess reliability, two trained rater reviewed 12 (26%) of the 46 cases (transcripts and Tracking Forms). The transcripts used for interrater reliability (IRR) assessment were the first 12 available transcripts from across the sites and therapists.

### Procedures

The data for this study included each transcript of the approximately 60-min audio-recorded DT interview and its accompanying 2-page Tracking Form (Additional file 1), which documented the steps of the intervention process. The first author, serving as the first auditor, accessed the data from a secure server at the University of Florida College of Nursing. Then she read and re-read each transcript in its entirety to get a feel for the overall sense of the interview. Once she had a broad sense of the dialogue, she reviewed the transcript to code each Adherence Checklist item per the Code Book definitions. The second auditor completed the same process to code the 12 transcripts selected for IRR evaluation. Following the statistical analysis for IRR, the two auditors met to resolve discrepancies through consensus to determine the final score for each item and refine the Code Book.

### Analysis

Descriptive statistics (mean, standard deviation, and ranges) were calculated for each of the Process items and the Core Principles items. Cohen’s kappa was calculated on the pre-consensus codes of the two auditors to evaluate the IRR for the Process items, which were scored dichotomously. For the Core Principles items, for which there was Likert-type scoring, weighted kappa was calculated to evaluate the IRR of the pre-consensus codes of the two auditors. Additionally, for the items that were Likert-type scale, Cronbach’s alpha was calculated to test for internal consistency of the ratings for the items. Descriptive statistics were also calculated across transcripts.

## Results

There was one partial transcript that was excluded because technical problems with the recording process prevented availability of a full transcript. Therefore, we reviewed 46 transcripts and each corresponding Tracking Form (hereafter referred to as transcripts): 30 sets were from DT interviews conducted by chaplains; and 16 sets were from DT interviews conducted by nurses. The number of transcripts per therapist ranged from two to ten.

### Reliability

For the 12 transcripts rated by 2 auditors, Cohen’s kappa and weighted kappa ranged between .75 and 1.0 (Tables 4 and 5) for all items. The mean Cohen’s kappa across the Process items was 0.97. The mean weighted kappa across the Core Principles items was 0.96. Fourteen scores (6%) required discussion to reach consensus. For the Core Principles items, Cronbach’s alpha was .92.

### Fidelity scores

The Process scores for the 46 transcripts ranged from 9 to 15. The mean Process score was 12.4/15 (SD = 1.2) (Table 6). Thirty-two transcripts (70%) had a mean Process score that met/exceeded 12, the minimum expectation for an 80% process fidelity score.

**Table 6:** DT Intervention Process Scores and Core Principles Scores Across Cases (*N* = 46)

	Process Score ^a^	Core Principles Score ^b^
**Mean Score**	12.4	9.9
**Median Score**	12.4	10.0
**Standard Deviation**	1.2	1.8
**Range**	3.6	4.8
**Min/Max**	10.4/14	7/11.8

*DT* Dignity Therapy

*Min/Max* Minimum/Maximum

^a^ Highest Possible Process Score = 15

^b^ Highest Possible Core Principle Score = 12

At a more granular level, regarding the frequency that each Process item was met, statistics appear in Table 4. There was perfect adherence to the process as evaluated by the first auditor (*n* = 46, 100%) for items 13, 14, and 15. These items focused on attention to the editing and review process. Lowest scores were noted in areas of verifying the individual’s understanding of the intervention purpose (*n* = 5, 11%) and the therapists’ consistency to keep the interview focused on creating the legacy document (*n* = 25, 54%). A simple ‘yes’ regarding understanding was common in the transcript, but not consistent with the training, which indicated the need for elaboration by the patient, and therefore was not scored as meeting the understanding item.

The Core Principles scores for the 46 transcripts ranged from 2 to 12. The mean Core Principles score was 9.9/12 (SD = 1.8) (Table 6). Thirty-two (70%) of the transcripts had a Core Principles score of 10 or higher, meeting the minimum expectation of an 80% score.

For the Core Principles items (Table 5), with a maximum score of 2 per item, the lowest mean score by item was 1.4 (SD = 0.6) in the area of maintaining the overall flow and pattern of the protocol. The highest mean score was 1.9 (SD = 0.4) for the therapists’ demonstration of behavior that supported patients’ dignity and for dialogue that connected with the individual beyond their illness or frailty.

## Discussion

In this study focused on describing the fidelity to the DT intervention by therapists trained according to the Refined DT Training Protocol [22], preliminary findings provide insights for maintaining and improving fidelity in delivery of the DT intervention. We identified indications that therapists had gained understanding and skills regarding the vital components of the intervention. We found that 70% had scores that were greater than 80% adherent to the original DT protocol. For the specific items, we found many strong consistencies in the delivery. Since 11 of the 15 items that related to process had a greater than 84% fidelity, and these were steps specific to the DT protocol, this offered a good indication that the therapists applied the process consistently. Mean scores for the Core Principles items revealed that the therapists generally had a better-than-moderate adherence with the primary essence of the deepest imperatives of the psychotherapeutic intervention. Again, as these items are specific to the DT model, this finding provided a good indication that the therapists implemented the model.

With regard to IRR, Cohen’s kappa and weighted kappa indicated there was excellent to perfect agreement across the sample of 12 transcripts between the two auditors [23]. Cronbach’s alpha demonstrated there was excellent internal consistency among the Core Principles items, as scored [24].

The process items that proved to be the greatest challenge for the therapists were related to having the participant verbalize their understanding of DT. This step is important so that the therapist redirects the participant back to the goal as needed during the course of the interview. Both issues indicate a need to remind the therapists that they must not only know the goal themselves, but verbally communicate the goal to the individual. It is noted, that some of the therapists asked individuals whether they understood the purpose of DT and accepted a ‘yes’ without requiring that the individual state clearly what they understood; in these cases, the therapist was not credited as having met the requirement to have the individual verbalize in their own words the purpose of the DT interview. The Core Principles item that caused the greatest challenge was the maintaining of the intended flow of the DT protocol. As with the process items that proved challenging, this item also required that the therapist overtly and intentionally move the process forward toward the end-goal of the legacy document. These findings are interesting in that they are consistent with common challenges seen in the medical environment where clinicians may be aware of the goals, but must intentionally communicate so that the individual (patient) is on the same track [25].

Although there is increasing attention to the imperative for intervention fidelity, there are no clear standards established [26, 27]. Further, the literature regarding fidelity in brief psychotherapies and communication interventions is more scant than in research regarding fidelity with medication or other physical treatments [1]. Some literature surveyed considered > 70% to be adequate [28] or high fidelity [27]; others considered 80% to be acceptable, or high fidelity or the minimum criterion below which remedial training would be required for interventionists [29]. Our findings have prompted us to examine how we used the Adherence Checklist and Code Book with a focus on understanding how to evaluate fidelity when so much content is in the subjective experience of communications between people on highly emotion-laden topics. This is in keeping with other researchers who have identified the need for clarification regarding intervention fidelity in psychotherapeutic and communication-based interventions [1]. Vital to this endeavor is the development of well-defined coding definitions that can inform all team members in implementing their roles, including attention to apparent and possible subjective experience. This focus includes those conducting the interviews, those providing guidance and mentoring, and those evaluating for intervention adherence. Code book definitions must include factors such as the frequency and extent to which categories are evident to participating parties in the communication [30]. Categories should be mutually exclusive to avoid confusion between categories and the code book should provide very specific instructions [30]. This type of code book should help the team member to have clarity such as how many times a statement or communication technique is needed. Future research is needed to complete this important work before DT is implemented in studies or in clinical practice.

As we examined the fidelity scores across the transcripts, we recognized that notable percentages of the therapists had mean scores that fell below the 80% minimum criterion for the Process scores and Core Principles scores. The range of scores was very wide for the Core Principles scores, creating concern that some therapists performed far below the minimal criterion, or possibly that our evaluations were failing to reflect some important elements in the process. The fact that therapists had as few as two interviews and as many as 10 leaves the question as to whether there is a cumulative effect regarding interviewing proficiency as therapists receive feedback about their performance. Since this study focused on the transcripts early in the trial, future fidelity evaluations will provide additional insights about the number of interviews typically needed to achieve sufficient proficiency needed for fidelity. Future research should focus on initial proficiency and drift from proficiency over the curse of a study to inform implementation and monitoring of DT delivered as part of clinical practice.

Several limitations are noted in our study. First, there were only two raters and both attended the training session together. Also, due to the small sample (46 total), the 26% subset that was reviewed for IRR only amounted to 12 transcripts. Further, all IRR comparisons were evaluations of transcripts early in the study. These limitations leave questions regarding whether such high levels of agreement would have been reached between additional or different coders and whether the primary coder would have experienced a drift in coding over time.

Another limitation was that the Likert-type scaled items were coded as None, Moderate, or Excellent, which did not allow for an evaluation that an item was determined as “Little” or “Minimal.” This created a vague definition in which an item that was met only minimally, may have been coded None by one rater, while the other rater coded the item as Moderate. For future coding, we recommend revising None to None/Insufficient as this change would provide greater clarity regarding the item definition. Future analysis of a larger sample is needed to explore the results over time as therapists gain benefit from experience and ongoing training and mentoring processes. The additional transcripts may allow for the emergence of improved scores as therapists increase in experience. Providing the fidelity scores to therapists strategically during group mentoring/support meetings may facilitate performance improvement by giving therapists insight regarding areas of inconsistency in their communication. When the intervention involves communication, the skill of the therapist is the treatment; therefore, the impact will be reliant upon the actual clarity and consistency of the communication. We anticipate that attending to these opportunities to fine tune processes will be important to ensure fidelity of the delivery of DT in our ongoing study, in which we foresee an additional 200 participants.

In conclusion, with Mean Process scores and Core Principles scores that exceeded the study criterion of 80% fidelity, we contend that the systematized training and evaluation of the transcripts provides preliminary evidence that merits further exploration. This study provides evidence of reliability for the DT Adherence Checklist, supporting its use in the analysis of the interview transcripts. We offer the Refined DT Training Protocol and the Adherence Checklist with the Code Book as important and useful tools to support intervention fidelity for researchers and clinicians implementing Dignity Therapy. With these considerations in mind, our results offer an important contribution to the understanding of fidelity in psychotherapy and communication-based interventions.

## Supplementary Information

**Additional file 1.**

## Data Availability

The datasets used and analyzed during the current study are available from the corresponding author on reasonable request.
