# Supplementary material for: Dignity therapy intervention fidelity: a cross-sectional descriptive study with older adult outpatients with cancer
Source: BMC Palliat Care. 2022 Jan 11;21:8. doi: 10.1186/s12904-021-00888-y (PMC8751346; doi:10.1186/s12904-021-00888-y)
Supplement: Supplementary file 1 — Additional file 1. [file 12904_2021_888_MOESM1_ESM.pdf]

Palliative Care for the Elderly

Dignity Therapy Contact & Process Tracking Form

<u>Contact #1 – Pre-Interview Call – By Phone:</u>

Patient Name		Today's Date (Phone Call)	
Phone Number		Name of Caller Today	
Email Address (optional)		Role of Above (RA, RN, Chaplain)	
Age		Confirm Consent has been Completed	Yes ☐ No ☐
Cancer – Diagnosis (Type)		Next Appointment (DT Interview)	
Date of Diagnosis			
Stage			

Before beginning, confirm patient's understanding about the Dignity Therapy interview:

- Do you know what Dignity Therapy is? (If no, say: **The purpose of Dignity Therapy is to create a written document with the thoughts and words that you want to share with your loved ones.**)
- Would creating a legacy document be meaningful to you at this time?
- Would you like me to review this with you before I proceed with my questions?

Goals for this call:

- Learn a little something about who the patient is (e.g., marital status; living arrangements, vocation; understanding of diagnosis; for whom their legacy document is intended)
- Inform the patient that the goal for the call is to review what the patient would want included in his/her legacy document
- Encourage the patient to think about the questions in advance of the session
- Remind the patient it is <u>not all about biography</u>; for many it is about <u>lessons learned</u>, <u>passing along wisdom</u>, <u>providing comfort to loved ones</u>, etc.

Learn from Patient:

1. What are your important goals of creating the legacy document?
2. Who are the family members in your immediate circle (names, relationships)?
3. Are there things I should be aware of that you hope to avoid speaking about in our conversation?
4. Confirm the patient has the list of Questions and orient him/her to think about the things he/she wants to address when you meet in person for the interview session—the important goals of creating this legacy document.
   Yes ☐ No ☐

©2020 DT Team

©

Palliative Care
for the Elderly

Contact #2 – DT Interview – In Person:

Update Contact Info			Today's Date (Interview)	
Patient prefers to receive final document via -	In person ☐ Mail ☐ Email ☐		Name of DT Therapist	
Does patient wish to designate another to receive their Legacy Document if they are not available when it is completed?	Yes ☐ No ☐		Role (RN, Chaplain)	
If Yes to above, note the Designee's Name, Relationship, Phone & Email				

Contact #3 – Review of Edited Draft Legacy Document – In Person:

Update Contact Info			Today's Date (Transcript Review)	
Editing completed per protocol	Yes ☐ No ☐		Name of DT Therapist	
Legacy Document read to patient?	All ☐ Part ☐ None ☐ If not read, note reason ________		Role (RN, Chaplain)	
Does the patient wish to make any changes to the document?	Yes ☐ No ☐			
If Yes, have changes been made and submitted to Amelia?	Yes ☐ No ☐ N/A ☐			

Contact #4 – Legacy Document Presentation – In Person, by Mail, or by Email:

Update Contact Info			Today's Date (Legacy Document Presentation)	
Format of Document	Hardcopy In Person ☐ Hardcopy by Mail ☐ Email ☐		Role of Above (RA, RN, Chaplain)	
Recipient of Document (Name & Relationship to Patient):				
Recipient's Address & Phone:				

©2020 DT Team
